# Sutureless management of left ventricle wall rupture; a series of three cases

**DOI:** 10.1186/s13019-014-0136-2

**Published:** 2014-09-02

**Authors:** Remco Bergman, Jayant S Jainandunsing, Bozena D Woltersom, Inez J den Hamer, Ehsan Natour

**Affiliations:** Department of Anesthesia and Pain Medicine, University Medical Center Groningen, University of Groningen, Hanzeplein 1, Groningen, 9700 RB The Netherlands; Department of Cardiothoracic Surgery, University Medical Center Groningen, University of Groningen, Hanzeplein 1, Groningen, 9700 RB The Netherlands

**Keywords:** Left ventricle wall rupture, Sutureless, Collagen sponges

## Abstract

Treatment of left ventricle wall rupture is very challenging, ruptured myocardial tissue is usually of poor quality and has a high risk of total rupture when being sutured. Furthermore, rapid decision-making is needed under stressful conditions. We present a series of three cases demonstrating the feasibility of using only hemostatic collagen sponges for the management of left ventricle wall rupture. All patients we Caucasian males, two patients were 65 years and one patient was 67 years old at the time of surgery. This report contains the first video images of solely use of hemostatic collagen sponges to seal a left ventricle wall rupture. Implication of our case series could be that the indication to use hemostatic collagen sponges, could be broadened towards other surgical specialties where suturing ruptured tissue can be difficult.

**Electronic supplementary material:**

The online version of this article (doi:10.1186/s13019-014-0136-2) contains supplementary material, which is available to authorized users.

## Background

Left ventricular free wall rupture (FWR) can be found in 26 percent of the cases in which patients die of acute myocardial infarction [1]. Overall incidence of FWR ranges from 4-6% [2]. Clinical course of FWR is highly variable but in most circumstances, it is a medical emergency mandating immediate treatment. Majority of patients surviving FWR had a contained ventricular wall rupture. Tissue surrounding the injury site is usually in poor condition and vulnerable to manipulation. Surgical techniques, such as suture or ligature can be sometimes ineffective in cases of poor tissue quality, increasing the risk of enlargement of the rupture. Short-term survival secondary to surgery can be as high as 76%, in patients with sub-acute FWR, long-term survival, however, is still poor in the end only 48.5% survive hospital stay [3].

## Case presentation

We present three patients who presented for emergency surgery for pericardial tamponade due to FWR. In two cases, FWR occurred secondary to a myocardial infarction. In both cases, diagnosis was confirmed by both trans-thoracic echocardiography (TTE) and pericardiocentesis. Our third case had an FWR as a complication during minimally invasive surgery, which was converted to a median sternotomy.

**Figure 1:**
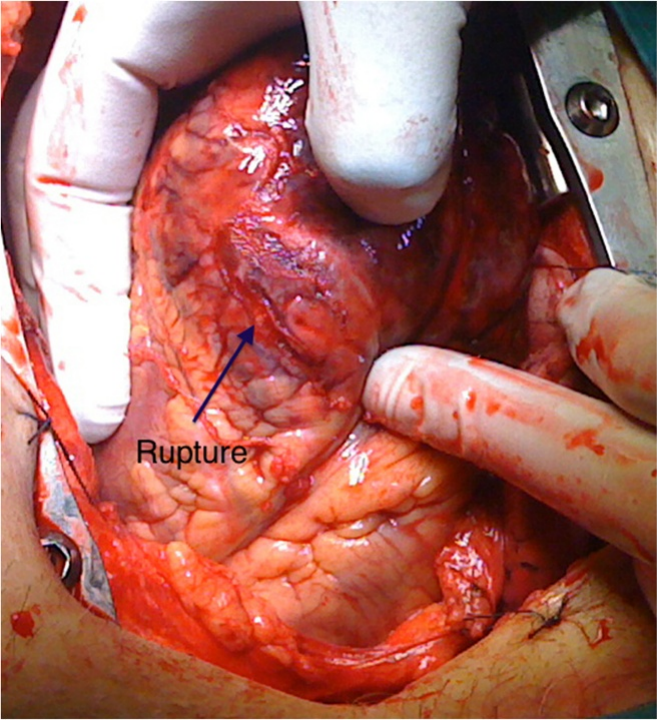
**A left ventricle wall rupture (arrow) can be seen, injury site is surrounded by bruised tissue.**

**Figure 2:**
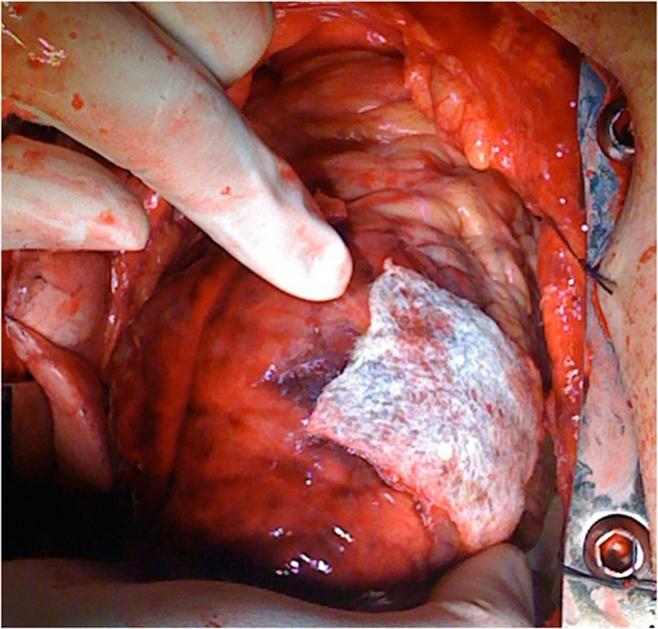
**Hemostasis in the injured area is achieved after application of the collagen sponge.**

Patient 1, a 67-year-old Caucasian male was operated under clinical conditions of a tamponade. He was referred for surgery after dissection of his left anterior descending (LAD) artery during a percutaneous coronary intervention (PCI). Indication for PCI was acute anterior wall infarction. Tissue quality in the injured area was deemed inadequate for suturing (Figure 1). Decision was made to use a collagen sponge (Tachosil®) to adhere the ruptured tissue and achieve hemostasis (Figure 2, Additional file 1).

Patient 2, a 65-year-old Caucasian male presented one week after a large anterior myocardial infarction with clinical signs of a cardiac tamponade. There was extensive infarcted tissue seen in the mid-range area of the LAD. A hemostatic collagen sponge (Tachosil®) was applied on the damaged tissue to stop bleeding. Subsequently, a fibrin sealant (Tissucol®) was used to adhere the injured area to the pericardium.

Patient 3, a 65-year-old Caucasian male underwent minimally invasive surgery for mitral valve repair. At the end of surgery after weaning off bypass severe bleeding from the pericardial region was noticed. The area of bleeding could not be clearly localized, a sternotomy was deemed necessary. Cardiac inspection revealed a rupture in the inferolateral wall. We used a hemostatic matrix sealant (Floseal®) to effectively stop bleeding and a collagen sponge (Tachosil®) to adhere the epicardium onto the injured area.

All patients survived treatment and in hospital stay, no reoperation was needed for bleeding. Routine transthoracic echocardiography months after surgery did not reveal any ventricle aneurysm formation.

## Conclusions

Our cases demonstrated the feasibility of using collagen sponges and hemostatic matrix sealant, for effective hemostatic sealing of ventricular free wall ruptures, even if tissue quality is poor.

Collagen sponges have been used in different surgical specialties ranging from urology, gynecology and liver surgery [4]-[6]. The use of collagen sponges in cardiac surgery is not new, its safety has been assessed in different cardiothoracic procedures [7]-[9]. However, the indication for using collagen sponges in ventricle wall rupture is becoming more common now, with similar good results using Tachosil®, for FWR [10]-[12]. The novelty in our case series was that one of our patients has an acute left ventricle wall rupture during a minimal invasive procedure not related to myocardial infarction. In this case time was of essence and the patch could be placed very rapidly with good results. Unlike other reports we did not encounter any complications within the first few months of follow-up [13],[14]. There are several issues associated with patients presenting with ventricular free wall rupture. First, these patients are usually presented as cardiac emergencies requiring immediate treatment. Second, the underlying culprit is most likely myocardial infarction implying poor ventricle function and third, emergency anticoagulation therapy is normally commenced on the base of an infarction before these patients arrive at a hospital, which further complicates intra-operative hemostasis. Free wall ruptures, especially in the occurrence of myocardial infarction, implies poor tissue quality, suturing infarction tissue makes it prone for further rupture.

The American Food and Drug Administration approved collagen sponges for use as an adjunct to hemostasis in cardiovascular surgery when control of bleeding by standard surgical techniques, such as suture, ligature or cautery, is ineffective or impractical. However, the sole use of collagen sponges and other hemostatic compounds for ventricular wall repair is an off-label use. Although other options exist to treat ventricle wall rupture, such as bioglue® or bovine pericardial patch, collagen sponges may be alternative in those centers where it is available.

Care should be taken to understand the caveats associated with the surgical approach emergency cases. When using bioglue® or bovine patches the patient should be on cardiopulmonary bypass with total decompression of the heart. The patch is then applied to a bloodless, motionless operative site and a “time to set” is needed before the heart is filled and ventricular contraction resumed. These extra minutes will ensure that the patch is firmly attached to the myocardial surface. In our cases the Tachosil® patch was applied directly on a beating heart, while two of the patients were not even on by-pass. The collagen sponge coated surface ensured direct attachment to the ventricle wall. Application of local pressure was of short duration, with good outcome results. Post-operative echocardiography demonstrated wall motion abnormality around the area of the rupture; however it did not demonstrate myocardial constriction related to the patch.

In case of ventricular free wall rupture, it is up to the surgeon to judge if repair can be undertaken using more conservative methods. However, if tissue quality makes a repair seem unlikely this new treatment method introduces a new option in operative management. Our cases have demonstrated that collagen sponges together with other hemostatic adjuncts can be a viable option as sole treatment for left ventricular bleeding, in patients with poor tissue quality, especially when there is limited time.

## Consent

Written informed consent was obtained from all patients for publication of this case report and any accompanying images. A copy of the written consent is available for review by the Editor-in-Chief of the journal of cardiothoracic surgery.

No Ethical approval was required, we used collagen patches in an emergency setting. The decision to use the patch was made on the spot.

Our patients were not part of any clinical trial, hence no ethical committee approval was required. The patches we used have CE markings and are approved for medical/surgical use in Europe and USA.

## Additional file

## Electronic supplementary material

Additional file 1: The video shows application of wound inspection of a LV rupture (patient 1) after application of a Tachosil® hemostatic sponge on a beating heart. The sponge is firmly adhered to the LV wall and no bleeding is seen. (mp4 358 KB)

Below are the links to the authors’ original submitted files for images.

Authors’ original file for figure 1

Authors’ original file for figure 2

## Abbreviations

FWR: Left ventricular free wall rupture
TTE: Trans-thoracic Echocardiography
PCI: Percutaneous coronary intervention
LAD: Left anterior descending coronary artery
