# Supplementary figures and images for: Sutureless management of left ventricle wall rupture; a series of three cases
Source: J Cardiothorac Surg. 2014 Sep 2;9:136. doi: 10.1186/s13019-014-0136-2 (PMC4177065; doi:10.1186/s13019-014-0136-2)

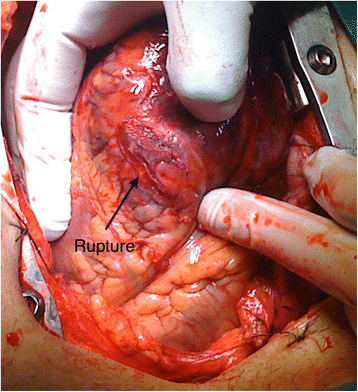

Supplement: Supplementary file 2 — Authors’ original file for figure 1 [file 13019_2014_136_MOESM2_ESM.gif]

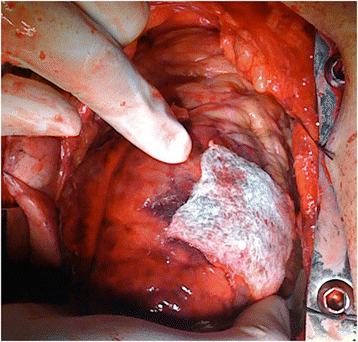

Supplement: Supplementary file 3 — Authors’ original file for figure 2 [file 13019_2014_136_MOESM3_ESM.gif]
